# Role of Non-Invasive Ventilation in Elderly Patients: Therapeutic Opportunity or Medical Futility? An Updated Narrative Review

**DOI:** 10.3390/medicina61071288

**Published:** 2025-07-17

**Authors:** Francesca Sangiovanni, Giulia Sartori, Nadia Castaldo, Alberto Fantin, Ernesto Crisafulli

**Affiliations:** 1 Respiratory Medicine Unit, Department of Medicine, Azienda Ospedaliera Universitaria Integrata of Verona, University of Verona, 37126 Verona, Italy; sangiovanni.1francesca@gmail.com (F.S.); giulia.sartori.verona@gmail.com (G.S.); af@albertofantin.com (A.F.); 2 Department of Pulmonology, S. Maria della Misericordia University Hospital, 33100 Udine, Italy; nadiacastaldo.nc@gmail.com

**Keywords:** acute respiratory failure, non-invasive ventilation, elderly, acute exacerbation of COPD, acute cardiogenic pulmonary oedema, community-acquired pneumonia, palliative care, end-of-life decisions, do not intubate order

## Abstract

*Background and Objectives*: Acute respiratory failure (ARF) represents an increasingly relevant clinical challenge in older subjects due to population aging and the high prevalence of cardiopulmonary comorbidities. Non-invasive ventilation (NIV), developed as continuous positive airway pressure (CPAP) or bilevel positive airway pressure (BiPAP), has become a first-line treatment in various forms of ARF, including acute cardiogenic pulmonary oedema (ACPE) and acute exacerbations of COPD (AECOPD), offering several clinical advantages. In this context, the limited evidence on the efficacy of NIV in older patients leaves considerable uncertainty as to whether it constitutes a valid therapeutic option or represents medical futility in these patients. *Materials and Methods*: This narrative review explores the use of NIV and its outcomes in four key clinical scenarios in the elderly: ARF due to ACPE, AECOPD, community-acquired pneumonia (CAP), and palliative/end-of-life care. *Results*: Strong evidence supports NIV use with improved outcomes in ACPE and AECOPD, even in older populations. Conversely, data on its use in pneumonia are inconclusive, with potential harm if applied inappropriately. In palliative care, NIV can help relieve symptoms, but if not used appropriately, it may extend suffering. *Conclusions*: Age alone does not appear to be a sufficient factor to determine whether or not to use NIV; it becomes relevant only when considered in conjunction with the purpose of its use and the patient’s clinical history and condition. Data remain limited and often conflicting, particularly when investigating the elderly population and patients with a “do not intubate” (DNI) order. There is a need for additional research on these patients, focusing on long-term outcomes and quality of life.

## 1. Introduction

In 1920, the “iron lung” was used for the first time to counteract the paralysis of respiratory muscles in children affected by the polio epidemic [1]. In the 1990s, non-invasive ventilation (NIV) was, for the first time, considered a mechanical therapeutic option for patients with acute respiratory failure (ARF) due to severe cardiogenic pulmonary oedema [2] and acute exacerbations of chronic obstructive pulmonary disease (COPD) [3]. Today, NIV is a first-line treatment for ARF from various aetiologies, such as COPD exacerbation, acute cardiogenic pulmonary oedema (ACPE) and weaning of acute exacerbations of COPD (AECOPD) patients from mechanical ventilation; weaker evidence also exists for aetiologies like pneumonia, asthma exacerbations, and acute respiratory distress syndrome (ARDS) [4,5]. In general, NIV has become an excellent support in recent years for reducing invasive mechanical ventilation (IMV) and, in particular, IMV-associated risks such as those connected with the need for endotracheal intubation (ETI), tracheostomy tube placement, or loss of normal airway defence mechanisms (which carries the risk of ventilator-associated pneumonia, prevalent in older patients) [6]. In addition, NIV allows for less sedation, allowing patients to drink, eat, communicate, and cough, reducing the work of respiratory muscles, improving gas exchange, and promoting alveolar recruitment. NIV significantly reduces these patients’ healthcare costs, length of stay, and mortality rates [7].

Although the efficacy of NIV is well proven, especially in some respiratory conditions, it has not been widely and specifically explored in elderly patients. This aspect is crucial due to the increasing incidence of ARF with age: elderly patients are often affected by chronic conditions, particularly cardiological and respiratory ones, which are the most common aetiologies underlying the development of ARF [8]. What is particularly complicated to define today is when NIV should be used and, more importantly, what the purpose of its use is in these patients: whether for therapeutic intent, palliative intent, or as maximum support in patients who have been designated as “do not intubate” (DNI). The literature on this topic is still limited and sometimes presents economic and ethical considerations that are difficult to clarify. Finally, therapeutic choices should consider extending life and the patient’s well-being, wishes, and quality of life [9]. In this narrative review, we have explored the updated scientific literature to clarify whether using the NIV in elderly patients with ARF in four different clinical scenarios may be a therapeutic opportunity or medical futility.

## 2. Definition and Leading Causes of ARF in Elderly Patients

It has long been known that the incidence of ARF increases exponentially with age, with an exponential increase each decade until age 85 years, with a particular growth peak from age 65 [10]. Indeed, with age, many changes occur in respiratory function: a decreased strength of the respiratory muscles, a decrease in the lung’s elastic recoil, and a decrease in chest wall compliance [11]. These changes lead to a reduction in expiratory flow rates, an increase in dead space and shunt fraction, and a reduction in diffusing capacity—it is estimated that the partial pressure of oxygen in arterial blood (PaO_2_) decreases by an average rate of 0.35 mmHg per year—with more requirement for increased minute ventilation during exercise or illness through increases in respiratory rate and work of breathing muscles (that have an age-related decrease in strength) [12]. The weakness of breathing muscles also leads to a reduced peak cough flow, facilitating acute respiratory tract infections. Moreover, elderly subjects have a significantly lower ventilatory response to both hypoxia and hypercapnia [13,14]. In addition, older adults are more likely to have concomitant illnesses, such as cardiovascular diseases, cerebrovascular accidents, delirium, renal and gastrointestinal diseases, and nutritional disorders, that have an impact on their respiratory system and that contribute significantly to respiratory decompensation in older adults [15,16].

We can, therefore, understand why it is much easier to develop ARF, defined as a deficit of the “lung” (gas exchange failure manifested with hypoxemia with normocapnia or hypocapnia) or of the “pump” (alveolar hypoventilation and hypercapnia) or both [17]. As demonstrated by Ray P. and colleagues, the leading causes of ARF in elderly patients (patients over 65 years old) are ACPE (43%), community-acquired pneumonia (CAP) (35%), AECOPD (32%), pulmonary embolism (18%), and acute asthma (3%). It is important to note that 47% of elderly patients with ARF had more than two clinical diseases [18].

## 3. Dimension and Relevance of the Problem

As to why the incidence of ARF increases exponentially with age, it is essential to note that the elderly population is growing steadily worldwide, along with life expectancy and the number of comorbidities among patients. Old patients (over 65 years) usually treated by the ED (emergency department) are estimated to be nearly 40% [19], in particular with an increased age admission rate [19]. Patients aged 75 years or older, also referred to as “very old patients”, represent 10–15% of intensive care unit (ICU) admissions [20]. Moreover, elderly patients have greater severity, a greater number of hospitalisations, and a greater risk of adverse events after discharge [21,22]. The Italian Emergency and Urgency Information System data analysis showed that in 2015, older adults over 75 accounted for 17% of ED visits and 62% of hospital admissions [23]. In the same study, ARF and acute-on-chronic respiratory failure were the most frequent discharge diagnoses from hospitals among persons aged 75 years or over, confirming the frequency of hospitalisations [23].

ARF is also a common complication in patients already hospitalised for other diagnoses, worsening their outcomes and mortality rate. A Brazilian study considering patients admitted to surgical and non-surgical ICUs reported the development of a subsequent ARF in 16%, which was a key contributing factor to their condition, with a poor prognosis observed particularly in older patients (more than 64 years) [24]. Considering older patients (mean 80 years) with ARF, the in-hospital mortality was explicitly reported as 16% [18], although it may reach up to 30% [25] to 31% [10], strongly conditioned by older age. A recent and extensive Italian study on retrospective administrative data on hospitalisations in Geriatrics Units found that in-hospital mortality or ICU transfer due to ARF was 27% [26]. Of note, particularly in older adults, patients who are intubated and mechanically ventilated may develop a condition defined as chronically critically ill [25,27], having devastating consequences for patients, families, and the entire health system due to the higher mortality, less quality of life once discharged with significant mental and physical outcomes, and an incredible amount of costs for families and the health system [28,29]. This aspect is relevant if considering patients with ARF, who represent the vast majority of patients with prolonged ICU stays [30]. Over time, these considerations need to be matched with the increased DNI orders with patient age (20% with age less than 60 years, 22% with age between 60 and 75 years, and 49% with age more than 75 years) and with the overall growth of the elderly population (from 2005 to 2009, the pooled rate of DNI was 19%; from 2010 to 2014, it was 26%; and from 2015 to 2019, it was 32%) [31]. In this context, NIV has significantly improved the survival of selected patients and reduced the severity of complications associated with IMV. Despite the rising incidence of ARF, NIV has contributed to a reduction in mortality [32].

In conclusion, clinicians may be faced with elderly patients who are remarkably fit and appropriate candidates for intubation or ICU stay, as well as individuals presenting with advanced or terminal cardiorespiratory conditions in which extension of life could not always be an appropriate goal, leading to the ethical problem of end-of-life decisions. Despite the magnitude of the situation at the clinical, economic, and social levels, there is no consensus specifying how and when to use NIV in elderly patients with ARF. Therefore, examining the existing literature on this topic is essential across the various clinical scenarios.

## 4. Clinical Scenarios and Indications for NIV

What initially seems to be most important when dealing with elderly patients is to determine whether the clinical situation requires a curative, palliative, or end-of-life approach, which is often very difficult. Subsequently, it is essential to understand what type of ARF we face to know the indications and the likelihood of NIV success and ensure no contraindications [33].

The 2017 European Respiratory Society (ERS)/American Thoracic Society (ATS) guidelines outline the clinical scenarios in which the use of NIV is supported by scientific evidence of efficacy [34]. In acute or acute on chronic respiratory acidosis due to AECOPD, without an immediate deterioration, BiPAP (bilevel positive airway pressure) can reduce the need for ETI and mortality [34]; other benefits were also provided by NIV, with different evidence for ARF due to ACPE (with both continuous positive airway pressure—CPAP and BiPAP [35]), for ARF in immunocompromised patients, for ARF resulting from post-surgical complications or chest trauma, for facilitating weaning from IMV in hypercapnic respiratory failure, and for palliative purposes in patients with end-stage cancer or terminal conditions [34]. In this context, however, due to limited studies, no recommendations or mention have been made on using NIV in older respiratory patients or its efficacy with increased age [34]. This aspect might lead to the non-defined and perhaps incorrect assumption that there are no differences in the use of NIV among the elderly population. Also, the British Thoracic Society (BTS)/Intensive Care Society (ICS) practice guidelines on hypercapnic ARF [36] and the ventilatory management report only mention the elderly population, defining a good outcome for patients with AECOPD aged more than 75 years in terms of intubation avoidance and reduced mortality with BiPAP [37], and in general satisfactory results [38].

There is little evidence on older patients. However, efficacy in general is also reported in ARF due to an immunocompromised condition (shorter periods of ventilatory assistance and ICU stays, fewer infectious complications, and lower ICU and hospital mortality, compared with IMV, in particular in less severe illness) [39,40,41], in ARF developed following surgical procedures or chest trauma [42,43,44,45], including predominantly younger patients. In contrast, although not by selected studies, some evidence appears in the older population when associated with hypercapnic ARF and concerning the context of NIV in weaning from IMV. In particular, a Chinese study involving 90 patients (mean age 68 years) requiring intubation and IMV for hypercapnic respiratory failure, which is exacerbated by pulmonary infection, early extubation followed by NIV demonstrated a reduced IMV duration, lower ventilator-associated pneumonia (VAP) risk, and decreased hospital mortality [46]. Similar results have been shown in a multicentre Italian study involving 50 patients (mean age 69 years) with severe hypercapnic ARF due to AECOPD in which NIV led to a shorter weaning time, reduced time spent in the ICU, decreased incidence of nosocomial pneumonia, and improved 60-day survival rates [47]. On the contrary, however, an extensive multicentre study performed in France and involving more than two hundred intubated patients for ARF (median age 70 years) observed no significant difference in the reintubation rate between patients weaned through the endotracheal tube, early extubation followed by NIV, or oxygen therapy [48].

From a clinical point of view, we will proceed to analyse the available scientific evidence concerning the application of NIV in the four disease scenarios, three primary causes of ARF in elderly patients (ACPE, AECOPD, and CAP), and finally, in the palliative or end-stage settings.

## 5. Disease Scenario #1: ACPE

While the medical management of ACPE is well established in clinical guidelines, the same cannot be reported for treating ARF secondary to this condition. The ERS/ATS guidelines [34] in the context of ACPE recommend using NIV with a “conditional recommendation and low certainty of evidence”. Similarly, the most recent European Society of Cardiology (ESC) guidelines provide a Class IIa recommendation for NIV, with a level of evidence B, in patients presenting with respiratory distress (respiratory rate > 25 breaths/min, pulse oximetry (SpO_2_) < 90%) [49]. The National Institute for Health and Care Excellence (NICE) recommends NIV in patients with severe dyspnoea and acidaemia [50]. Considering that the average age of patients with ARF due to heart failure is approximately 70 years [51], it is reasonable to assume that the results obtained from the studies can be applied to an elderly population.

Although a most extensive randomised study (more than one thousand patients) conducted on NIV in patients with ARF due to heart failure was carried out on an elderly population (mean age of 78 ± 10 years) and reported a benefit of BiPAP and CPAP in terms of improved dyspnoea and respiratory distress, the same did not demonstrate a significant difference in short-term mortality or need for ETI compared to the standard therapy group [52]. Of note, a risk of bias may be possible due to the switching of approximately 15% of patients from the standard therapy group to the BiPAP group [52]. Different results concerning lower hospital mortality, intubation rate, and shorter ICU length of stay with a more rapid resolution of respiratory symptoms and improved patient comfort in ventilated patients compared to standard medical care have been reported [53,54]. In particular, a meta-analysis including 34 randomised trials and more than three thousand patients (mean age from 64 to 77 years) showed that BiPAP or CPAP significantly reduced mortality and the need for IMV [55]; furthermore, BiPAP was associated with a more rapid improvement in dyspnoea and hypercapnia [55]. In the context of ED, the randomised study by Crane and colleagues [56] demonstrated in acidotic ACPE (mean age 75 years) the superiority of CPAP in terms of survival compared to BiPAP and standard treatment (ST).

Furthermore, the study by L’Her and colleagues [57], which compared CPAP with ST in a selected population aged more than 75 years, showed the superiority of CPAP in reducing mortality (7% vs. 24%) only in the first 48 h. Finally, the study by Moritz and colleagues [58] found equal efficacy of BiPAP and CPAP in reducing respiratory distress with similar clinical outcomes in patients with a mean age of 78 years for both treatments.

The absence of a clear superiority of CPAP over BiPAP, or vice versa, may be attributed to the central role played by positive end-expiratory pressure (PEEP) in this clinical context; regardless of whether it is delivered via CPAP or BiPAP, its beneficial effects are primarily attributable to the reduction in respiratory workload and effort, as well as a decrease in preload and an improvement in ventricular performance through the mitigation of volume overload [59,60]. These findings have led to the widespread use of BiPAP and CPAP in this clinical scenario, even in the elderly. A recent real-life multicentre prospective study (mean age 81 years old) highlighted that in 80% of patients with ARF due to heart failure (in-hospital mortality rate of 9%), BiPAP and CPAP were used as first-line therapies and that one-quarter of patients initially treated with standard oxygen therapy were switched to BiPAP or CPAP [35]. Of note, in the same study, the initial treatment with oxygen therapy showed an odds ratio (OR) for treatment failure of 3.65 (95% confidence interval, CI 2.55 to 5.23), and the age of patients was not an independent predictor of clinical failure [35].

Finally, a French study also focused on the use of BiPAP in very elderly patients (>85 years old), demonstrating that when BiPAP is used for ACPE or AECOPD, these patients have similar hospital survival rates compared to younger patients, with an acceptable 6-month outcome. Instead, BiPAP in the context of DNI was associated with a poor outcome in both very old and younger patients [20].

Based on the current evidence, it can be concluded that overall findings tend to favour its efficacy regarding the use of BiPAP and CPAP in ARF due to ACPE (Table 1). Given the epidemiological context of the disease, these results can generally be applied to the elderly population, with no significant differences in very old patients. Nonetheless, further studies are warranted, particularly focusing on very elderly patients with a high burden of comorbidities and DNI patients.

## 6. Disease Scenario #2: Severe AECOPD

Very recent data have shown that the global prevalence of COPD is around 12%, rising to 24% in the population over 70 [78]; in this context, the prevalence of ICU admission rates ranges from 2 to 19% among all AECOPD cases requiring hospitalisation [79,80]. Age is a factor related to the prognosis of hospitalised AECOPD [81], especially in the early period from admission [82]. In these patients, using NIV also strongly predicts prognosis and hospital readmission [83]. However, the same NIV, among treatments, is supported by substantial evidence of their efficacy [84], primarily when AECOPD is associated with acute respiratory acidosis [61], which, if present at admission, more than doubles the risk of a prolonged hospital stay of more than seven days [85]. Operator-related factors may influence the outcome of NIV [86]. Focusing specifically on elderly patients, the data are somewhat conflicting.

Nava and colleagues, comparing outcomes achieved with BiPAP versus ST in patients over 75 years old (mean age 81 ± 3 years) with hypercapnic ARF (primarily due to AECOPD), found favouring results concerning BiPAP in terms of survival (in-hospital, at 6 and 12 months), reduction in the need for ETI, and reduction in tachypnea and hypercapnia [37]. Moreover, the sample analysed included many patients with DNR “do not resuscitate” orders, making it a somewhat representative sample of the real-world clinical profile of patients with AECOPD. BiPAP also proved to be an excellent rescue therapy for those initially treated with ST who later met the criteria for ETI, with a success rate of 75% in these cases [37]. Finally, the same study [37] demonstrated that patients treated with BiPAP had significantly shorter hospital stays (mean 19 days vs. 22 days for the BiPAP and ST groups, respectively), not considering patients switching from ST to BiPAP [37].

Nonetheless, questions remain regarding whether an upper age limit exists beyond which NIV is particularly indicated and which prognostic factors may reliably predict therapeutic success. A single-centre Turkish study conducted on 162 patients (≥65 years old) to carry out an age- and aetiology-based analysis of the clinical efficacy of BiPAP in hypercapnic ARF, considering mainly AECOPD, ACPE, and CAP, but also bronchiectasis and kyphoscoliosis, showed that in-hospital outcome differences were independent of age and instead dependent on the aetiology of ARF (again confirming the efficacy of BiPAP in patients with AECOPD and ACPE) [62]. Furthermore, the authors found that dyspnoea level, Glasgow Coma Scale (GCS), and APACHE-II (Acute Physiologic Assessment and Chronic Health Evaluation II) scores were independent predictors of BiPAP failure in AECOPD patients, in line with findings from several other studies [63]. Again, the in-hospital mortality was similar between patients older than 75 and those younger than 75, such as the intubation rate in the context of BiPAP failure [64]. Although the presence of comorbidities and the severity of the respiratory condition at admission were strongly associated with mortality, interestingly, 180 days after discharge, survival was significantly higher in the younger group [64]. This finding of lower long-term survival after discharge for the elderly population has been confirmed by two further studies [65,66]. However, another critical negative prognostic factor is the continued occurrence of AECOPD when each episode worsens the overall prognosis [67]. In this context, another role that NIV can play is in the chronic, home-care setting, helping to reduce hospital admissions, alleviate dyspnoea, and improve quality of life [87]; however, data regarding the acute management of these patients are limited.

As previously mentioned, another issue concerns elderly patients who, due to advanced age or multiple comorbidities, have a DNI order. In this context, a noteworthy study investigated acute-on-chronic hypercapnic ARF in a cohort of DNI patients over 75 who were already receiving long-term oxygen therapy [68]. The findings confirmed the effectiveness of BiPAP in this population, with a reported mortality rate of 13% [68]; however, age seems to be a critical factor, and the median age of BiPAP non-responders was significantly higher compared to that of responders (87 vs. 80 years) [68]. Notably, survival was favourable in the short term and over time, with 69% of patients alive at one year and 54% at three years, particularly when BiPAP was continued at home [69]. These results contrast markedly with those reported in a previous study, in which patients with a DNI order had a dramatically reduced in-hospital survival compared to non-DNI patients (26% vs. 74%) [70]. Specifically, among COPD patients, outcomes were slightly better. However, they followed the same trend (33% vs. 90%), with a progressive decline in survival over the following months, aligning with data highlighting the poor prognosis associated with repeated exacerbations [71]. Therefore, mortality in non-DNI COPD patients appears to be around 10%, rising sharply in the presence of a DNI order.

The evidence thus seems to confirm the effectiveness of NIV (in particular BiPAP) in treating hypercapnic acute respiratory failure secondary to AECOPD in elderly patients (Table 1). Its efficacy in DNI patients, however, remains less clear. It is important to emphasise that although it is well established that exacerbations have an essential impact on the health economy and worsen patients’ quality of life on physical, social, and psychological levels [88,89], no studies to date have specifically addressed the quality of life about age in this patient population (Table 2).

## 7. Disease Scenario #3: CAP

The use of NIV for pneumonia increased by 50% from 2000 to 2009 [90] despite the absence of specific recommendations supporting its use in this population in major clinical guidelines [91,92,93]. This rise in utilisation is likely attributable to findings from certain studies that demonstrated a reduction in intubation rates with NIV in patients with pneumonia [94,95], thereby extending the potential indication for NIV to this clinical scenario. A significant factor contributing to the widespread adoption of NIV in recent years has been the coronavirus disease 19 (COVID-19) pandemic. During this period, NIV was extensively utilised to manage moderate to severe ARF, primarily because it could be administered outside the ICU, which frequently operates at full capacity. Although its efficacy was sometimes limited, NIV emerged as a valuable therapeutic option [96]; in some cases, a success rate exceeding 60% has been reported [97]. Notably, a retrospective Italian study involving 79 patients with SARS-CoV-2 infection demonstrated that NIV could prevent intubation in nearly half of the cases [98]. Persistent symptoms, prevalently neurological with axonal damage [99,100] and lung function and ventilatory alterations [101,102,103] remained after infection, when, according to the form of disease suffered, the days of hospitalisation required, and other associated comorbidities have been reported [104]. Nevertheless, advanced age, prolonged duration of NIV prior to intubation, and the presence of neurological comorbidities have been identified as risk factors for adverse outcomes and increased mortality [105,106]. Moreover, it should be considered that NIV failure leading to intubation appears to be associated with higher mortality and more complications compared to IMV alone, which is also caused by delayed intubation [107,108].

Advanced age appears to be a risk factor for increased mortality in these patients [72]. Studies conducted in the elderly population do not show a mortality benefit of NIV over IMV, as Valley T.S. and colleagues highlighted in an extensive retrospective study on elderly patients [73]; however, NIV is associated with lower hospital costs. Nonetheless, NIV should not be chosen over IMV in elderly patients merely because it is less expensive, as some studies [74,75] have shown that first-line use of NIV can even be harmful if used in patients with severe non-respiratory organ dysfunction and very severe hypoxemia. In Al-Rajhi and colleagues’ study [74], hemodynamic instability was the strongest negative predictor of BiPAP failure.

In this context, a recent study [76] on very elderly patients (more than 80 years old) who were critically ill due to pneumonia found that NIV was not more effective than IMV in terms of mortality. Still, neither was it harmful, except in cases where the P/F ratio (PaO_2_/fraction of inspired oxygen-FiO_2_) was less than 150.

A recent study [77] specifically investigated how to closely monitor elderly patients with pneumonia undergoing NIV to avoid delaying intubation if necessary, suggesting careful monitoring of heart rate and arterial blood gases between 30 min and 2 h after initiating NIV.

Lastly, it is essential to consider the presence of three closely interconnected risk factors for NIV failure: delirium [109], which is a frequent condition, particularly in elderly patients undergoing NIV, the subsequent administration of benzodiazepines, and the resulting decreased level of consciousness, sometimes caused also by hypercapnia. This combination significantly increases the risk of aspiration. Aspiration, in turn, may further compromise respiratory function and contribute to clinical deterioration, thereby reducing the chances of NIV success [109,110]. Strong and conclusive data on using NIV in ARF due to pneumonia are lacking, both in the general population and in older and significantly older patients (Table 1).

## 8. Disease Scenario #4: Palliative Care

In 2017, a dedicated Task Force on the “Palliation Use of NIV” clarified that this mechanical support can be used for three purposes: (a) as life support with no preset limitations on life-sustaining treatments; (b) as life support when patients and families have decided to forego ETI; (c) as a palliative measure when patients and families have chosen to forego all life support, receiving only comfort measures [111].

The distinction among these scenarios lies in the intended goal of NIV. In the first two categories, NIV is utilised to improve gas exchange and provide a temporal bridge for treating acute causes of respiratory failure. In such cases, even if occasionally poorly tolerated, NIV should be encouraged [112]. Conversely, when the objective is palliative, the primary focus shifts to symptom relief. If NIV is poorly tolerated in this setting, it should not be promoted, as it may not provide meaningful comfort at the end of life [113]. In this last case, the risk lies in prolonging the patient’s life, thereby exposing him to unnecessary suffering and resulting in suboptimal use of medical and economic resources [114].

Dyspnoea is the predominant symptom in end-stage respiratory diseases, occurring in approximately 70% of terminal oncology patients and up to 90% of those with end-stage COPD [115]. However, particularly in elderly patients with advanced COPD, accurately predicting prognosis or identifying the terminal stage remains challenging [116]. This uncertainty complicates decisions regarding initiating palliative NIV and further impairs establishing effective and clear communication with patients, who are often unaware of the palliative intent of ventilatory support [117].

Unfortunately, the majority of studies evaluating NIV in COPD populations have focused on clinical endpoints, such as a reduction in intubation rates or mortality, rather than symptom control [118]. A review by Smith and colleagues [119] highlighted the limited evidence supporting the efficacy of NIV in relieving dyspnoea during AECOPD. A more recent publication [120] also noted the paucity of studies addressing the use of NIV for palliative purposes in COPD, attributing this gap both to the traditional emphasis of palliative care on neoplastic diseases and to the inherent difficulty in predicting disease trajectories in severe COPD with ARF. More robust data are available regarding using NIV in conjunction with home oxygen therapy in stable severe COPD patients [121].

On the other hand, good results have been seen with NIV in patients with solid tumours at the end of life [122,123,124].

## 9. Discussion

ARF is an increasing clinical and socio-economic burden due to population ageing and the high prevalence of comorbidities in older adults. In this context, the use of NIV, now considered first-line therapy in various clinical scenarios, is sometimes controversial or insufficiently studied in the elderly. Solid evidence supports its use in clinical scenarios such as ACPE and AECOPD, primarily due to the epidemiological predominance of these conditions in elderly cohorts. In these two scenarios, studies involving elderly patients, including those with DNI orders, suggest that NIV can improve survival, reduce the need for endotracheal intubation, and allow symptom control. Despite this, limited research has been conducted to identify risk factors that, beyond in-hospital mortality, are associated with poor long-term survival and quality of life, essential data to understand whether NIV is appropriate or simply a means of prolonging a person’s suffering, with particular attention on DNI and very old patients. Notably, there is a complete absence of randomised studies focusing on clinical and patient-centred benefits after hospital discharge (Table 1), resulting in scarce evidence supporting a true long-term benefit of NIV in the elderly population. Excluding the approach of symptoms, in this context, NIV may be seen as a medical futility.

Regarding ARF due to pneumonia, data on NIV use in the general population remain conflicting, and even more so in the elderly. Although some retrospective studies report reduced intubation rates, others highlight increased risks of NIV failure, especially in the presence of severe hypoxemia, hemodynamic instability, or delayed intubation. Advanced age, neurological comorbidities, and delirium are significant predictors of poor outcomes. Therefore, careful patient selection and close monitoring during the early phases of NIV are essential to maximise benefits and avoid harm. In this setting, the boundaries for NIV use remain poorly defined, underscoring the need for randomised controlled trials to provide robust evidence guiding the selection of elderly patients suitable for NIV.

Finally, when dealing with elderly patients, it is often necessary to consider important clinical and ethical decisions, such as end-of-life care. NIV may confer symptomatic relief of dyspnoea and enhance comfort in patients with end-stage respiratory diseases, notably those with advanced COPD or malignancies. Nevertheless, when NIV is poorly tolerated or risks prolonging suffering without demonstrable symptomatic benefit, its use should not be pursued. The extant literature reveals a lack of consensus and insufficient data about NIV application within palliative care for non-oncologic patients, warranting further research to inform clinical practice in this sensitive domain. In this regard, more in-depth literature is needed to clarify such a complex condition. Effective communication regarding therapeutic goals is paramount, given that patients and families may not invariably comprehend the palliative intent underpinning NIV deployment.

In any case, age alone does not appear to be a sufficient factor to determine whether or not to use NIV; it becomes relevant only when considered in conjunction with the purpose of its use and the patient’s clinical history and condition. This review accentuates the pressing need for high-quality, targeted studies focusing on elderly populations, particularly long-term outcomes, post-discharge quality of life, and shared decision-making processes, especially in advanced age and DNI status. The ethical implications must be rigorously considered: the provision of NIV should be predicated on clinical appropriateness and anticipated benefit, rather than solely on age, economic factors, or presumptions of futility.

## 10. Conclusions

Although current evidence remains limited and sometimes conflicting, NIV represents a potentially life-saving and cost-effective intervention in elderly patients with ARF, as long as it is used in the proper clinical context and with clear treatment goals. Age “a priori” should not be seen as a strict contraindication to NIV but as one factor to consider within a broader clinical, functional, and ethical evaluation. Future research should focus more specifically on elderly populations, prioritising clinically relevant outcomes such as functional recovery, health-related quality of life, and patient-centred metrics to find the key to making the best use of NIV in this heterogeneous and progressively expanding patient population.

## Figures and Tables

**Table 1:** Original studies examining the use of NIV during different acute clinical scenarios in elderly patients.

First Author and Year	Nationality of Study	Study Design	Total Patients	Mean Age of Patients (y)	Clinical Scenario	DNI (%)	Study Design	Main Results
Gray A., 2008 [52]	UK	RCT	1156	78	ACPE	NS	ST vs. CPAP vs. BiPAP	BiPAP and CPAP are superior to ST only in reducing symptoms and hypercapnia
Crane S.D., 2004 [56]	UK	RCT	60	75	ACPE	NS	CPAP + ST or BiPAP + ST vs. ST	CPAP + ST is superior to BiPAP + ST and ST in in-hospital survival
L′Her E., 2004 [57]	France	RCT	89	84	ACPE	NS	ST vs. ST + CPAP	ST + CPAP is superior to ST but only reduces early 48-h mortality
Moritz F., 2007 [58]	France	RCT	109	78	ACPE	NS	ST + CPAP vs. ST + BiPAP	ST + CPAP is equal to ST + BiPAP
Aliberti S., 2018 [35]	Italy	POS	1293	81	ACPE	NS	CPAP or BiPAP vs. ST	BiPAP or CPAP is superior to ST
Schortgen F., 2012 [20]	France	POS	1019	2 groups: <80 y and ≥80 y	Multiple: ACPE, AECOPD, de novo ARF, post-extubation, DNI	31% and 34%	BiPAP in >80 and <80 years old	BiPAP in ≥80 y patients is equal to BiPAP in <80 y patients in the context of AECOPD, ACPE and post-extubation
Plant P. K., 2000 [61]	UK	RCT	236	69	Mildly and moderately acidotic AECOPD	NS	BiPAP vs. ST	BiPAP reduces the need for ETI and in-hospital mortality compared to ST
Nava S., 2011 [37]	Italy	RCT	82	81	AHRF (mostly AECOPD)	75%	BiPAP vs. ST	BiPAP is superior to SMT in decreasing the rate of meeting the ETI criteria and the mortality rate
Çiftci F., 2017 [62]	Turkey	POS	162	≥65	Multiple: AECOPD, ACPE, CAP, bronchiectasis, kyphoscoliosis	NS	BiPAP in different ages	The efficacy of BiPAP is independent of age. BiPAP is most successful in COPD or ACPE.
Phua J., 2005 [63]	Singapore	POS	111	COPD (72 y) and non-COPD (67 y)	AHRF	NS	BiPAP in COPD patients and non-COPD	BiPAP is more effective in preventing ETI in AHRF due to COPD than in non-COPD conditions. A high APACHE II score predicted BiPAP failure in both groups
Nicolini A., 2014 [64]	Italy	POS	207	2 groups: ≥75 y and <75 y	COPD	10% of >75 years old	BiPAP in different ages	Age is not a determining factor for outcomes.
Titlestad I.L., 2013 [65]	Denmark	RA	253	72	COPD	10%	Long-term survival in patients treated with BiPAP due to AECOPD	30-day mortality was 24%, and 5-year survival was 23.1%. Age and DNI are conditioning factors
Lindenauer P. K., 2018 [66]	USA	RA	2,340,637	≥65	AECOPD	NS	Long-term survival after hospitalisation due to AECOPD	Discharge from the hospital is associated with prolonged risks of readmission and death. Age and DNI are conditioning factors.
Ankjærgaard K. L., 2017 [67]	Denmark	RA	201	71	COPD	71%	Risk of readmissions and death after BiPAP and discharge	Poor prognosis
Scarpazza P., 2008 [68]	Italy	POS	62	81	Acute on chronic ARF (mainly COPD)	100%	Short-term outcomes after BiPAP in DNI	Successful
Scarpazza P., 2011 [69]	Italy	RA	52	≥75 y	Acute on chronic ARF (mainly COPD)	100%	Long-term outcomes after BiPAP in DNI	Successful
Fernandez R., 2007 [70]	Spain	POS	233	2 groups: DNI (73.8), not-DNI (67.1)	Multiple: AECOPD, ACPE, CAP, others	15%	BiPAP in DNI and not-DNI for different aetiologies	BiPAP offers lower expectations in DNI. COPD is associated with a better short-term prognosis
Chu C. M., 2004 [71]	China	POS	107	73.2 ± 7.6	AECOPD	32%	Long-term outcomes after BiPAP	High risk of readmissions and death. DNI patients have worse outcomes.
Carrillo A., 2012 [72]	Spain	POS	184	2 groups: de novo ARF (62 y), non de novo (72 y)	CAP	NS	Risk factors for BiPAP failure	Successful BiPAP is associated with better survival. If predictors for NIV failure are present, avoiding delayed ETI could minimise mortality. Older age independently predicts hospital mortality.
Valley T.S., 2004 [73]	USA	RA	12.480	64	CAP	NS	NIV vs. IMV in 30-day mortality	There are no differences in 30-day mortality
Al-Rajhi A., 2018 [74]	Canada	RA	218	73	CAP	0%	NIV failure and outcome of NIV vs. IMV	Predictors of NIV failure: need for hemodynamic support, younger age, low burden of comorbidities and lower PaO2/FiO2
Antonelli M., 2001 [75]	Europe and the US	POS	354	58	Hypoxemic ARF	0%	Risks of NIV failure	Predictors of NIV failure: older age, high severity score, CAP and ARDS
Besen B.A.M. P., 2021 [76]	Brazil	RA	369	86	CAP	NS	NIV vs. IMV	NIV is not superior to IMV in terms of hospital mortality
Park M. J., 2020 [77]	South Korea	POS	78	77	ARF	NS	Risks of NIV failure	Pneumonia at admission is a risk factor for NIV failure. Age is not a conditioning factor for NIV failure in pneumonia

Abbreviations: POS indicates prospective observational study; RA, retrospective analysis; RCT, randomised controlled trial; ACPE, acute cardiogenic pulmonary oedema; AECOPD, acute exacerbation of chronic obstructive pulmonary disease; AHRF, acute hypercapnic respiratory failure; ARDS, acute respiratory distress syndrome ARF, acute respiratory failure; BiPAP, bilevel positive airway pressure; CAP, community-acquired pneumonia; CPAP, continuous positive airway pressure; DNI, do not intubate; ETI, endotracheal intubation; IMV, invasive mechanical ventilation; NIV, non-invasive ventilation; ST, standard therapy; NS, not specified.

**Table 2:** Outcomes analysed.

Author and Year	Outcomes								
	In-Hospital Mortality	Symptoms During Hospitalisation	Intubation Rate	Mortality After Discharge (Months)			Length of Hospital Stay	Quality of Life After Discharge	Readmission Rate
				1	3–6	12 or More			
Gray A., 2008 [52]	**✓**	**✓**	**✓**	**✓**			**✓**		
Crane S.D., 2004 [56]	**✓**	**✓**	**✓**						
L′Her E., 2004 [57]	**✓**	**✓**	**✓**				**✓**		
Moritz F., 2007 [58]	**✓**		**✓**				**✓**		
Aliberti S., 2018 [35]	**✓**		**✓**						
Schortgen F., 2012 [20]	**✓**		**✓**		**✓**		**✓**	**✓**	
Plant P. K., 2000 [61]	**✓**	**✓**	**✓**				**✓**		
Nava S., 2011 [37]	**✓**	**✓**	**✓**				**✓**		
Çiftci F., 2017 [62]	**✓**	**✓**	**✓**						
Phua J., 2005 [63]	**✓**		**✓**				**✓**		
Nicolini A., 2014 [64]	**✓**		**✓**		**✓**		**✓**		
Titlestad I.L., 2013 [65]	**✓**			**✓**		**✓**			
Lindenauer P. K., 2018 [66]						**✓**			**✓**
Ankjærgaard K. L., 2017 [67]	**✓**					**✓**			**✓**
Scarpazza P., 2008 [68]	**✓**	**✓**					**✓**		
Scarpazza P., 2011 [69]						**✓**			**✓**
Fernandez R., 2007 [70]	**✓**		**✓**	**✓**	**✓**		**✓**		
Chu C. M., 2004 [71]						**✓**			**✓**
Carrillo A., 2012 [72]	**✓**		**✓**				**✓**		
Valley T. S., 2004 [73]	**✓**		**✓**	**✓**					
Al-Rajhi A., 2018 [74]	**✓**		**✓**				**✓**		
Antonelli M., 2001 [75]	**✓**		**✓**				**✓**		
Besen B. A. M. P., 2021 [76]	**✓**		**✓**						
Park M. J., 2020 [77]	**✓**	**✓**	**✓**				**✓**		

Abbreviations: Checkmark “**✓**” indicates that the outcome in question has been analyzed.

## Data Availability

Not applicable.
